# Accelerated Brain Aging in Multiple Sclerosis: Microstructural and Metabolic Correlates of the Brain Age Gap

**DOI:** 10.3390/neurolint18070124

**Published:** 2026-06-29

**Authors:** Anas Z. Nourelden, Fen Bao, Abigail Biddix, Nidhi Patel, Mawadda Abdelhai, Basil Memon, Vivian Truong, Zaima Liaquat, Carla Santiago-Martinez, Yongsheng Chen, Anza B. Memon

**Affiliations:** 1 Department of Neurology, School of Medicine, Wayne State University, Detroit, MI 48201, USA; fbao@med.wayne.edu (F.B.); hu5060@wayne.edu (A.B.); hr1043@wayne.edu (N.P.); hu7557@wayne.edu (M.A.); basil.memon@gmail.com (B.M.); vtruong@wayne.edu (V.T.); zaimaliaquat@wayne.edu (Z.L.); csantiago@med.wayne.edu (C.S.-M.); ys.chen@wayne.edu (Y.C.); eg6818@wayne.edu (A.B.M.); 2 College of Literature, Science, and the Arts, University of Michigan, Ann Arbor, MI 48109, USA; 3 Department of Neurology, John D. Dingell, Veterans Affairs Medical Center, Detroit, MI 48201, USA

**Keywords:** multiple sclerosis, brain age gap, aging, neurodegeneration, MRI, brain atrophy, N-acetylaspartate

## Abstract

Background/Objectives: Multiple sclerosis (MS) can cause neurodegeneration leading to accelerated brain atrophy. Brain-predicted age (BA) is an emerging neuroimaging biomarker for neurodegeneration but remains underexplored in MS. This study examines the pathophysiological substrates associated with the brain age gap in MS compared with healthy controls (HCs) through a combination of volumetric, spectroscopic, and diffusion imaging. Methods: This retrospective cross-sectional study included 33 HCs and 124 MS patients. Participants underwent 3T MRI including 3D-T1, MR spectroscopy, magnetization transfer, and diffusion imaging. BA and volumes were estimated from T1-weighted scans using brainageR. Metabolic integrity (total N-acetylaspartate to total creatine ratio, tNAA/tCr) and microstructural damage (magnetization transfer ratio [MTR], fractional anisotropy [FA]) were evaluated independently in normal-appearing tissues. Multivariate linear regression assessed MS diagnosis as an independent predictor of BA metrics, controlling for age, sex, and race. Results: MS patients showed significantly higher predicted brain age (53.3 vs. 31.8 years) and a markedly larger age gap (10.2 vs. −0.1 years) compared to HCs. Beyond macroscopic volume loss, accelerated aging paralleled profound subclinical degradation, including lower neuronal integrity (tNAA/tCr: 2.0 vs. 2.4) and widespread microstructural damage, evidenced by reduced MTR and FA across both normal-appearing gray and white matter. Linear regression confirmed MS diagnosis as an independent predictor of both BA and Age Gap (15.09 and 13.50 years) after adjusting for confounders. Conclusions: MS patients exhibit accelerated biological brain aging, characterized by a significant age gap and concurrent tissue volume loss. The brain age gap in MS extends beyond macroscopic atrophy, capturing underlying subclinical metabolic failure and widespread microstructural degradation in normal-appearing tissues. This positions BA as a robust, multi-dimensional proxy for neuroaxonal pathology.

## 1. Introduction

Multiple sclerosis (MS) is a chronic, immune-mediated inflammatory demyelinating and neurodegenerative disease of the central nervous system, characterized by focal inflammatory demyelination, progressive neuro-axonal loss, and the accumulation of lesions throughout both white and gray matter [1,2,3]. Approximately 85–90% of patients initially present with relapsing-remitting MS (RRMS), characterized by acute neurological dysfunction followed by periods of remission and potential recovery [4,5]. Early intervention is important, as subtle brain atrophy and microstructural damage in normal-appearing white matter (NAWM) can be detected even at the earliest disease stages [6,7,8]. Prompt initiation of effective therapy reduces relapse rates and mitigates silent disability accrual and long-term functional decline [3,9]. High-efficacy disease-modifying therapies (DMTs) have been associated with a younger brain age, where predicted biological age falls below chronological age. Longitudinal data demonstrate that MS patients in early disease state who remain clinically stable exhibit significantly slower annual brain age progression compared to those experiencing disability worsening [10,11].

Predicted brain age is an imaging biomarker derived from machine learning to quantify neuroanatomical deviations from healthy aging trajectories by estimating the biological age of the brain from routine MRI data [8,10,12]. In MS, this biological age consistently exceeds chronological age, a phenomenon termed the brain age gap (BAG) that signals accelerated neurodegeneration [7,13]. The magnitude of this gap may range from 4.21 to over 10 years in RRMS cohorts, whereas secondary-progressive patients can reach values as high as +13.3 years [11,12,14]. Brain aging in MS progresses approximately 15% faster than chronological aging, with one study documenting a mean annual increase of 1.15 brain years per calendar year [11]. This accelerated aging correlates with disease burden, showing associations with white matter lesion volume and global atrophy measures [15,16].

A core structural signature associated with higher predicted brain age is periventricular morphological changes, such as enlargement of the lateral, third, and fourth ventricles, coupled with insular atrophy [8]. Advanced diffusion imaging reveals that disorganization within commissural fibers, specifically the forceps major and minor, emerges as a core microstructural correlate of both increased disability and accelerated brain aging [17]. Tract-specific abnormalities further distinguish progressive disease, with elevated radial diffusivity and reduced radial kurtosis in the corticospinal tract and anterior thalamic radiation strongly linked to elevated brain age [17]. At the lesional level, paramagnetic rim lesions (PRL) serve as predictors of advanced brain aging, conferring an additional 5.6-year increase in BAG compared to PRL-negative individuals [9].

Metabolic dysfunction complements these structural findings. Reductions in N-acetyl aspartate (NAA), a neuronal mitochondrial metabolite and proxy for neuro-axonal integrity, represent a pivotal subclinical change in MS [4,18]. This NAA decline manifests within focal lesions and normal-appearing gray matter, preceding measurable parenchymal loss [6,18]. Such metabolic shifts correlate with brain age by encoding the cumulative burden of neuronal injury and energy deficiency, which deep learning models interpret as a premature aging phenotype [7,16].

As a surrogate biomarker, BAG predicts future disability accumulation, with higher baseline values forecasting shorter time to Expanded Disability Status Scale (EDSS) progression [10,15]. The metric also captures cognitive dysfunction, correlating strongly with processing speed on the Symbol Digit Modalities Test [11,19]. In early MS, the annualized increase in brain age helps identify patients at imminent risk for decline, as those with worsening disability exhibit significantly greater annual changes than stable individuals [11]. These properties position brain age as a promising trial endpoint for evaluating neuroprotective or remyelinating therapies [12,13]. Recent large-scale studies have robustly established the BAG as a significant biomarker for disability accumulation and disease progression in MS. For instance, longitudinal analyses have demonstrated that BAG can predict EDSS worsening and cognitive decline, often exceeding the predictive value of traditional volumetric measures. However, while these large-cohort studies confirm the presence of accelerated aging, they rely predominantly on structural T1-weighted MRI, leaving the underlying metabolic and microstructural substrates of this ‘biological gap’ largely unexplored. Understanding whether the brain age metric captures these subclinical pathological shifts is crucial for its validation as a comprehensive biomarker.

Additionally, most brain age algorithms suffer from systematic regression to the mean bias, consistently overestimating age in younger subjects and underestimating it in older cohorts [20,21,22]. In addition, unmeasured confounders, including lifestyle factors such as smoking and alcohol use, cardiovascular comorbidities, and demographic variables, can independently influence BAG, obscuring disease-specific neurodegeneration [8,13,19]. These limitations necessitate rigorous multivariate approaches that control for potential confounders while simultaneously investigating the microstructural and metabolic substrates underlying the brain age gap. Therefore, our study aimed to quantify the magnitude of accelerated brain aging in MS compared with normal controls using a multimodal neuroimaging framework while controlling for chronological age, gender, race, and body mass index. Also, by utilizing a multi-modal framework, incorporating MR spectroscopy (MRS), magnetization transfer imaging (MTI), and diffusion tensor imaging (DTI), we sought to determine how accelerated biological aging reflects metabolic failure and microstructural damage in normal-appearing brain tissues, while controlling for key demographic and clinical confounders.

## 2. Materials and Methods

### 2.1. Participant Recruitment and Selection Criteria

We conducted a retrospective cross-sectional analysis of patients with MS and healthy volunteers. Eligible participants with MS were aged 18–60 years, had a diagnosis confirmed by a board-certified neurologist and MS specialist/neuroimmunologist using the 2017 McDonald criteria, and had been free of clinical relapses and systemic corticosteroids for ≥30 days before imaging. Treatments initiated before enrollment were restricted to fingolimod, glatiramer acetate, natalizumab, or ocrelizumab. Exclusion criteria encompassed pregnancy and any concomitant neurological or psychiatric disorder (e.g., major depression or anxiety disorders). Data were extracted from the electronic medical records. This study was conducted in accordance with the ethical standards of the institutional research committee and with the 1964 Helsinki Declaration and its later amendments. The study protocol was approved by the Wayne State University Institutional Review Board (IRB# 01-66-12M1E). At the time of the original data collection, written informed consent was obtained from all participants, which included provisions for the secondary use of data for future research. Consequently, the requirement for additional patient consent for this specific retrospective analysis was waived. The authors assert that all procedures contributing to this work comply with the ethical standards of the relevant national and institutional guides. All patient data were anonymized, and no identifiable information is included in this manuscript.

### 2.2. Clinical Data

Demographic and lifestyle variables were recorded for all participants, including chronological age, gender, race, and body mass index (BMI). Behavioral factors, specifically smoking status and alcohol use, were also documented. For patients with MS, the clinical course (Relapsing-Remitting vs. Progressive forms) was recorded. Additionally, clinical disability (EDSS) and disease duration were documented for MS patients where available.

### 2.3. MRI Image Acquisition

Imaging was performed on a 3-Tesla Siemens Verio scanner (Siemens Medical Systems, Erlangen, Germany) utilizing a 12-channel radiofrequency (RF) head coil. The acquisition protocol began with a T2-weighted fluid attenuated inversion recovery (FLAIR) sequence (46 contiguous axial slices; repetition time (TR)/echo time (TE) = 9000/128 ms; inversion time (TI) = 2500 ms; 150° flip angle; 256 × 192 matrix; 1 × 1 × 3 mm^3^ voxel size), followed by T2-weighted turbo spin-echo imaging (46 slices; TR/TE = 7810/97 ms; 120° flip angle; 640 × 480 matrix; 0.4 × 0.4 × 3 mm^3^ voxel size). Structural assessment utilized a 3D T1-weighted Magnetization Prepared Rapid Gradient Echo (MPRAGE) sequence (176 slices; TR/TE = 1680/3.52 ms; TI = 900 ms; 9° flip angle; 384 × 384 matrix; 0.7 × 0.7 × 1.3 mm^3^ voxel size). For Magnetization Transfer (MT), gradient-recalled echo (GRE) images were obtained with and without a saturation pulse (1200 Hz off-resonance; 500° nominal flip angle; 10 ms Gaussian-apodized single-lobe sinc pulse), using TR/TE = 1200/3.64 ms, a 15° flip angle, and a 256 × 256 matrix with 1 × 1 × 3 mm^3^ resolution over 46 slices. Diffusion Tensor Imaging (DTI) was conducted via a single-shot spin-echo EPI sequence with a balanced Icosa21 scheme (20 directions; b = 1000 s/mm^2^; 2 averages; TR/TE = 10,400/126 ms; 90° flip angle; 200 × 200 matrix; 1.28 × 1.28 × 3 mm^3^ voxel size; 46 slices). Additionally, T1-weighted spin-echo scans were performed pre- and post-contrast (TR/TE = 400/4.47 ms; 80° flip angle; 256 × 256 matrix; 1 × 1 × 3 mm^3^ voxel size; 46 slices), although healthy controls received no gadolinium. Finally, ^1^H-MRS data were collected from a volume of interest (VOI) in the central white matter rostral to the lateral ventricles using a 2D multi-voxel Point-Resolved Spectroscopy (PRESS) sequence (TR/TE = 1500/135 ms; 8 averages; 50 Hz water suppression bandwidth; 16 × 16 chemical shift imaging (CSI) matrix; 8 × 8 voxel VOI; 10 × 10 × 15 mm/1.5 mL voxel size).

### 2.4. MRI Data Processing and Analysis

All scans were visually inspected for motion and ghosting artefacts. Subsequent post-processing and quality control utilized Jim software (version 6, Xinapse Systems Ltd., Essex, UK). The specific analysis pipelines were as follows:

#### 2.4.1. Brain Age Analysis

Brain-predicted age was estimated from raw 3D T1-weighted MPRAGE images using brainageR (version 2.1, University College London, London, UK) [23]. This R-based software employs Gaussian Process Regression using kernlab package. Images were segmented into gray matter (GM), white matter (WM), and cerebrospinal fluid (CSF) via Statistical Parametric Mapping 12 (SPM12, Wellcome Centre for Human Neuroimaging, London, UK), with segmentation accuracy verified visually using FMRIB Software Library (FSL, version 6.0.7.13, Oxford Center for Integrative Neuroimaging, Oxford, UK). Normalized probability maps were vectorized to predict age based on a model trained on 3377 healthy controls. The brain-predicted age gap (BAG) represents the difference between predicted and chronological age.

#### 2.4.2. Lesion Analysis

T2 hyperintense lesion volume (T2LV) was quantified in patients with MS using Jim software. Lesions were identified on T2-weighted turbo spin-echo images (referenced to FLAIR images) using semi-automated edge detection, with final verification by a certified neurologist.

#### 2.4.3. MT Ratio (MTR) Analysis

Following coregistration based on mutual information, MTR maps were generated using Jim software. MTR was defined as 100 × (MToff − MTon)/MToff. Mean MTR values were extracted for normal-appearing gray matter (NAGM) and white matter (NAWM) using tissue and lesion masks.

#### 2.4.4. DTI Analysis

Fractional anisotropy (FA) and mean diffusivity (MD) maps were created using DTI Studio (version 3.0, Johns Hopkins University, Baltimore, MD, USA). Mean FA and MD values were calculated for NAGM and NAWM, applying the same masking strategy used for MTR.

#### 2.4.5. Cortical Thickness and Volumetric Analysis

Cortical reconstruction and segmentation were performed on lesion-filled T1-weighted images using FreeSurfer (version 7.2, Athinoula A. Martinos Center for Biomedical Imaging, Charlestown, MA, USA). Parcellation relied on the Desikan-Killiany atlas. Cortical thickness (CTh) was measured as the distance between the pial and white-matter surfaces. Total deep GM volume included the thalamus, caudate, putamen, pallidum, hippocampus, amygdala, and accumbens. Choroid plexus volume (CPV) was normalized to total intracranial volume (TIV) to derive a normalized ratio (nCPV = CPV/TIV × 1000).

#### 2.4.6. MRS Analysis

Multivoxel ^1^H-MRS data were analyzed via LCModel (version 6.3, LCModel Inc., Oakville, ON, Canada) to determine the (NAA + NAAG)/(Cr + PCr) ratio within an 8 × 8 voxel volume of interest in central white matter. Estimates were generated using a custom basis set. To minimize non-brain tissue effects, voxels with Cramer–Rao bounds >20% were excluded. The mean ratio of valid voxels was calculated per participant.

### 2.5. Statistical Analysis

Data analysis was conducted using R (version 4.3, R Foundation for Statistical Computing, Vienna, Austria) and Jamovi (version 2.5, Jamovi project, Sydney, Australia). We tested continuous variables for normality via the Shapiro–Wilk test. Normally distributed data were reported as mean (standard deviation) and compared with Student’s *t*-tests, while non-normal data were reported as median (interquartile range) and compared using Mann–Whitney U tests. Categorical data were analyzed using Chi-square tests. For MS patients, the association between BA, BAG and clinical metrics (EDSS, disease duration) was evaluated using Spearman’s rho correlation coefficients. To identify independent associations between MS diagnosis and variables such as Age Gap, Brain Predicted Age, and Choroid Plexus Volume, we employed multivariate linear regression, adjusting for race, gender, age, and BMI. We checked for heteroscedasticity using the Breusch-Pagan test and multicollinearity using variance inflation factors (VIF), setting significance at *p* < 0.05. Group-level voxel-wise Z-score maps of MTR were generated by comparing MTR values in the MS group against the matched healthy-control distribution at each voxel, overlaid on anatomical templates in MNI152 space. Maps were computed separately for NAWM and NAGM and thresholded at *p* < 0.05 with a minimum cluster size of 100 voxels.

## 3. Results

### 3.1. Participant Demographics and Baseline Characteristics

The study included 157 participants, comprising 124 MS patients and 33 healthy controls (HCs). The overall cohort averaged 39.7 ± 11.2 years in age, with 65.0% being female. As detailed in Table 1, significant demographic differences were observed between groups. MS patients were significantly older (Median: 39.0 years, interquartile range (IQR) [32.0 to 48.0]) than HCs (Median: 30.0 years, IQR [27.0 to 40.0], *p* = 0.001). There were also significant differences in the distribution of race (*p* < 0.01) and smoking status (*p* = 0.01) between the MS and HC cohorts. No significant differences were found in gender (66.1% female in MS vs. 60.6% in HC, *p* = 0.700), BMI (*p* = 0.263), or alcohol use (*p* = 0.07). The majority of MS patients had RRMS (94.2%). MS patients had a median EDSS of 2.5, IQR [1.5 to 4], with a disease duration of 7 years (IQR [2 to 11 years]).

### 3.2. Accelerated Brain Aging and Neurodegeneration

**Figure 1:**
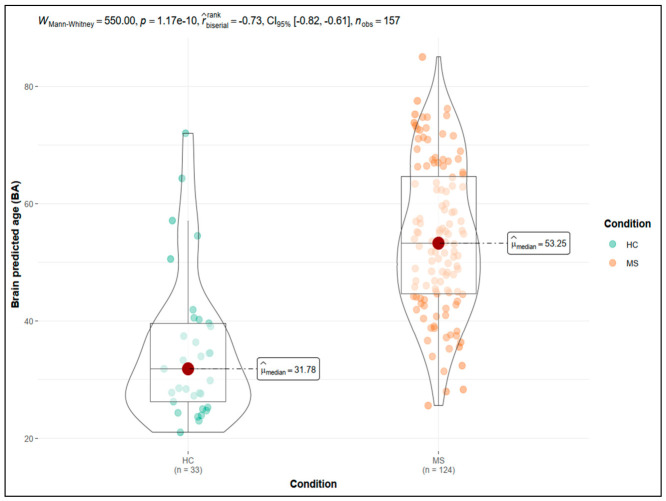
Violin plot comparing brain-predicted age between healthy controls (HC) and multiple sclerosis (MS) patients. The MS group exhibits significantly higher brain age compared to controls (*p* < 0.001). The central line in the violin plots represents the median.

**Figure 2:**
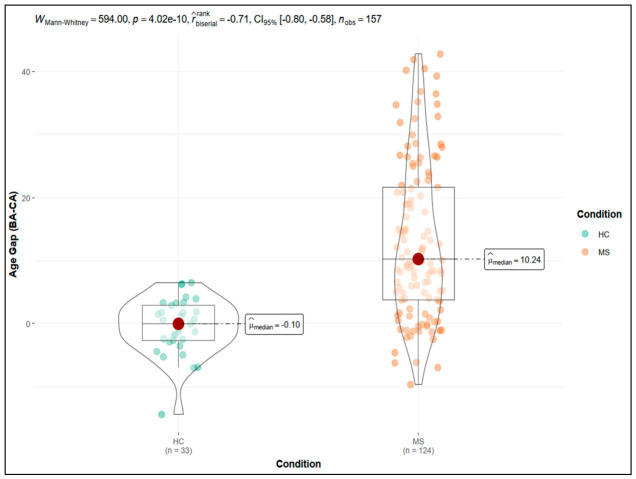
Violin plots comparing the Brain Age Gap (Brain Predicted Age minus Chronological Age). The MS group exhibits significantly higher brain age gap compared to controls (*p* < 0.001). The central line in the violin plots represents the median.

**Figure 3:**
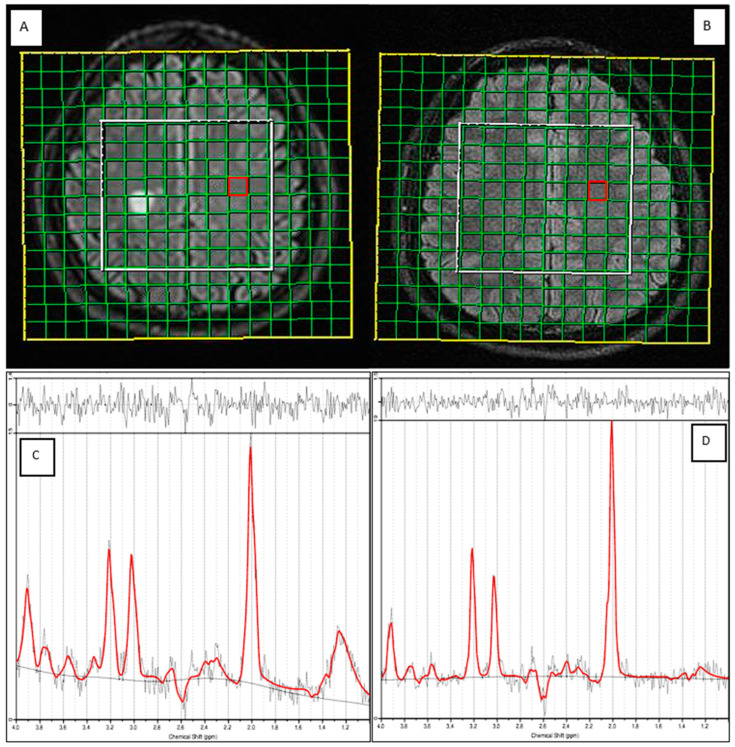
Representative ^1^H-MR spectra from a single voxel of the multi-voxel CSI acquisition in an MS patient (**A**,**C**) and a healthy control (**B**,**D**). (**A**,**B**) Axial localizer showing the chemical-shift imaging grid (yellow box) and volume of interest (white box); the analyzed voxel is marked in red. (**C**,**D**) Corresponding LCModel-fitted spectra (red line) with fit residual shown above. Major peaks include NAA and Cr at 2 parts per million (ppm) and 3 ppm.

**Figure 4:**
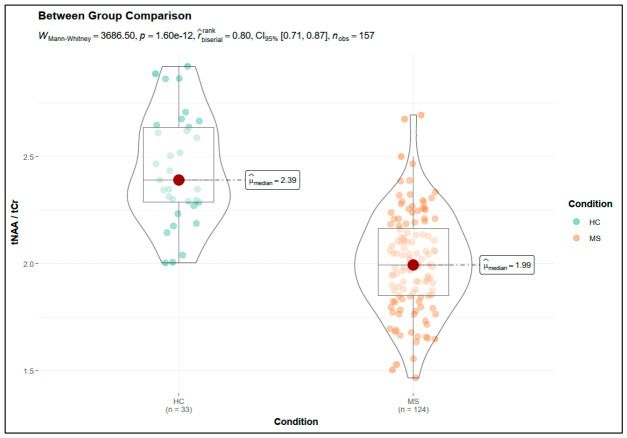
Violin plots comparing tNAA/tCr ratio. The tNAA/tCr ratio was significantly lower in MS than in HC. Boxes show the median and interquartile range; the violin shows the distribution, and individual data points are overlaid.

**Figure 5:**
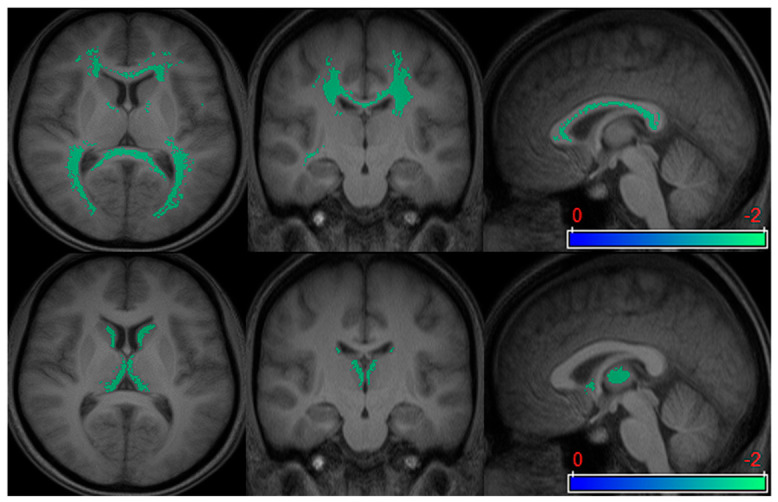
Z-score maps of MTR in NAWM (**top row**) and NAGM (**bottom row**) from 13 MS patients and 13 healthy controls, matched for age, sex, and race, were overlaid on an anatomical template in MNI152 space. Each map is shown in three orthogonal views, with the left, middle, and right columns corresponding to axial, coronal, and sagittal views, respectively, and a common Z-score color bar. Cool colors (blue/green) indicate regions with significantly reduced MTR values in MS patients compared with healthy controls (*p* < 0.05, cluster size = 100).

The MS cohort exhibited significant evidence of accelerated brain aging and structural atrophy compared to HCs, as summarized in Table 2 and visualized in Figure 1 and Figure 2. Representative spectra are shown in Figure 3 and the group tNAA/tCr comparison in Figure 4. Spatial mapping confirmed the distribution of these microstructural abnormalities, with significantly reduced MTR in the normal-appearing white matter and grey matter of MS patients relative to matched controls (Figure 5).

### 3.3. Multivariate Validation of MS Effect on Brain Aging

Multivariate linear regression analysis, presented in Table 3, confirmed that the MS diagnosis was an independent and robust predictor of accelerated brain aging, even after controlling for potential confounders (chronological age, gender, and race). The model explained a substantial proportion of the variance (R^2^ = 0.507). MS condition significantly predicted BA, with MS patients exhibiting an average 15.093 years older BA compared to HCs (95% CI [10.66, 19.53], *p* < 0.001). Another linear regression model evaluated the effect of MS diagnosis on Age Gap, adjusting for gender and race. The model explained 20.7% of the variance (R^2^ = 0.207), and MS diagnosis was a significant predictor of accelerated brain aging. The presence of MS increased the Age Gap by approximately 13.5 years relative to controls (95% CI [8.84, 18.16], *p* < 0.001). Gender and race were not significant predictors in this model. Additionally, higher BA and BAG were associated with higher EDSS (rho = 0.413, *p* < 0.001; rho = 0.273, *p* = 0.003, respectively), with BA also significantly correlating with disease duration (rho = 0.483, *p* < 0.001). The regression model for Choroid Plexus volume explained minimal variance (R^2^ = 0.058). MS diagnosis was not a significant predictor of choroid volume (*p* = 0.526). However, higher BMI was significantly associated with reduced Choroid Plexus Volume (Estimate: −0.009, 95% CI: −0.017, −0.001, *p* = 0.023).

## 4. Discussion

This study of 157 participants (124 MS patients, 33 healthy controls) demonstrated significant accelerated brain aging in MS, with patients exhibiting a median brain age gap of +10.2 years compared to −0.1 years in controls (*p* < 0.001). Multivariate regression confirmed MS diagnosis as an independent predictor of brain predicted age, with patients’ brains appearing approximately 15 years older than controls after adjusting for chronological age, gender, and race (Estimate = 15.09, *p* < 0.001). This accelerated aging was accompanied by widespread neurodegeneration, including 12.0% lower gray matter volume, 7.9% lower white matter volume, reduced cortical thickness, and impaired microstructural integrity in normal-appearing brain tissue. In contrast to all parenchymal measures, CSF volume was higher, reflecting ex-vacuo expansion of CSF spaces that accompanies tissue loss, consistent with the atrophic pattern underlying the accelerated brain aging. Choroid plexus volume showed no significant differences between groups (*p* = 0.097).

While recent large-scale and longitudinal studies have robustly established that BAG predicts clinical worsening and disability accumulation in MS [10,16], these investigations predominantly rely on macroscopic structural MRI. The core novelty of our study lies in translating this macroscopic ‘age gap’ into its subclinical pathophysiological substrates. Our multimodal findings suggest that a higher BAG is not merely a reflection of lost volume, but a proxy for underlying metabolic failure (reduced tNAA/tCr) and widespread structural disorganization (reduced MTR and FA) in tissues that appear radiologically normal. Our study demonstrated a significantly reduced MTR in NAGM (*p* = 0.006) and a reduced FA in NAWM (*p* = 0.031). These findings align with Caranova et al., who described microstructural alterations in normal-appearing tissues as a hallmark of multiple sclerosis that extends beyond visible lesions [1]. The reduced MTR we observed in NAGM is consistent with Ge et al., who observed lower MTR values in non-lesional tissue compared to healthy controls [6]. Furthermore, our observation of reduced FA in NAWM mirrors the typical Diffusion Tensor Imaging (DTI) profile of increased mean diffusivity and decreased anisotropy [2]. Newer diffusion models have identified decreased Neurite Density Index and lower Orientation Dispersion Index in pre-lesional tissues [1]. The lower MTR in our cohort suggests compromised tissue integrity, indicative of both inflammation and irreversible axonal loss [24,25]. This reduction often occurs independently of visible plaques [6]. Likewise, the DTI abnormalities we identified in the NAWM imply a breakdown of structural barriers within white matter tracts [6]. This means that the accelerated brain aging we observed is driven by invisible pathology that predates evident parenchyma loss [8,18].

Our observation of a significantly reduced tNAA/tCr ratio in MS patients (*p* < 0.001) suggests that the BAG captures axonal loss and metabolic failure rather than inflammation alone. N-acetylaspartate (NAA) is a well-established marker of neuronal health and metabolic activity [18,26]. Its reduction is interpreted as an indicator of axonal dysfunction and loss, which correlates more strongly with patients’ disability accumulation and cognitive impairment than demyelinating activity alone or lesion load [4,18]. NAA levels can fluctuate and partially recover during the resolution of acute edema or metabolic stress [6,18]. Conversely, the consistent, widespread reduction observed in chronic MS cohorts signifies permanent neuronal loss and serves as a harbinger of future atrophy [4,18]. A previous study by Ge et al. indicated that whole-brain NAA loss proceeds at a rate up to 3.6 times faster than brain volume loss [6]. Moreover, significant decreases in NAA have been shown to precede overt parenchymal atrophy [18]. This temporal dissociation implies that the Brain Age metric, when informed by MRS and microstructural data, detects neuroaxonal pathology before it manifests as irreversible gross atrophy [5,6].

Our findings corroborate existing literature demonstrating that accelerated brain aging is a fundamental feature of multiple sclerosis. Prior studies have shown substantial heterogeneity in its magnitude: for example, a large multicenter longitudinal study reported a mean BAG of +10.3 years in patients with MS compared with +4.3 years in healthy controls, exceeding estimates reported in dementia (approximately 9 years) and traumatic brain injury (approximately 4.7 years) [12]. Our observed median gap of +10.2 years (IQR 3.8–21.7; mean ± SD = 13.1 ± 12.3 years) aligns with this multicenter estimate; the wide interquartile range and large SD reflect between-patient heterogeneity. Other cohorts report less pronounced differences; a deep learning study documented a BAG of 6.98 ± 7.18 years [8], whereas two cross-sectional analyses have found mean gaps of 4.4 ± 6.6 years and 4.21 ± 6.51 years [7,11]. Matching our Age Gap estimate, a Chinese cohort reported a mean BAG of 13.0 ± 14.7 years in RRMS [14].

In a study by Romme et al., partial correlation analysis, adjusting for chronological age and sex, revealed a significant association between BAG and Expanded Disability Status Scale (EDSS) scores (ρ = 0.206, *p* = 0.004) [8]. This finding is in line with Cole et al., who asserted that a higher BAG led to greater disability, with every 0.64-year increase corresponding to a one-point EDSS elevation (*p* < 0.001) [12]. Longitudinally, the authors found that baseline BAG predicts EDSS worsening (Hazard Ratio 1.02; 95% CI = 1.01–1.03; *p* < 0.001) [12]. As later noted by Pontillo et al., annualized EDSS changes correlated directly with BAG progression (r = 0.48, 95% CI 0.36–0.58, *p* < 0.001) [16]. Structural BAGs also correlate with lesion burden; however, the predictive relationship may attenuate when controlling for normalized brain volume [10,12].

Smoking was reported among MS patients but in none of the healthy controls (*p* = 0.01). Current literature confirms that smoking is an independent modifier of brain health that can accelerate brain aging in MS [27,28]. Smoking has been linked to lower total white matter and deep gray matter volumes, independent of cardiovascular disease [28]. A study employing deep learning has quantified this impact, estimating that smokers’ brains appear approximately 1.5 years older than those of non-smokers [19]. Furthermore, smoking correlates with increased T2 lesion accumulation and greater disability accrual over time [28]. Correspondingly, another significant lifestyle factor is obesity, which is a consistent predictor of accelerated brain aging and lower gray matter volume [19]. These factors exhibit a synergistic adverse effect; the interaction between smoking and obesity has been shown to amplify the risk of cognitive decline and worsening physical disability beyond their additive burdens [27]. These lifestyle factors contribute to the neurodegenerative milieu; nevertheless, the magnitude of the BAG observed in our study far exceeds the estimated 1.5-year increase attributed to smoking alone [19]. While the MS group was older and included more smokers than controls, the chronological-age imbalance is removed by construction in the age gap, and the brain-predicted-age model was additionally adjusted for age; however, smoking was not entered as a covariate because all of the included HCs were non-smokers, so residual confounding from smoking and other unmeasured lifestyle factors cannot be excluded. Furthermore, as smoking is estimated to add only ~1.5 brain-years, it is unlikely to account for the ~13.5-year adjusted gap; nonetheless, our estimates should be interpreted as upper-bound associations. This suggests that lifestyle factors may modify the trajectory, yet the disease process remains the primary driver of accelerated aging. This aligns with findings that disease-related disability accumulation is a more powerful contributor to brain aging than clinical course alone [10]. Ultimately, neurodegeneration in MS likely emanates from the interplay between chronic disease pathology and co-occurring lifestyle modifiers [3].

Our study has several strengths, including the use of a multi-modal imaging approach that combines volumetric data with microstructural (DTI, MTR) and metabolic (MRS) markers, providing a comprehensive view of brain aging. Furthermore, the use of a robust regression model enabled us to isolate the effect of MS from potential confounders, such as age, sex, and BMI. However, some limitations must be acknowledged. First, the retrospective cross-sectional design precludes establishing a causal relationship between disease progression and accelerated aging over time. In addition, disease duration and EDSS could not be included as covariates in the regression models. Second, the relatively small sample size of the healthy control group compared to the MS cohort may affect the statistical power of certain analyses. Finally, our cohort was not stratified by MS phenotypes, and the small number of progressive cases precluded a subtype-stratified analysis. As 94.2% of patients had RRMS, our findings apply primarily to relapsing-remitting disease and should not be extrapolated to primary- or secondary-progressive phenotypes, whose neurodegenerative and aging trajectories may differ substantially. Future research should employ longitudinal designs to track the progression of the Age Gap, evaluate its sensitivity to DMTs, and try to isolate the effect of disease duration from that of the diagnosis itself. Additionally, integrating deep learning models that combine MRI with cognitive assessments (e.g., BICAMS), disability measures (e.g., EDSS), and other patient-reported outcome measures (PROMs) could enhance the predictive accuracy of the brain-age metric. Exploring the impact of specific comorbidities, lifestyle interventions and longitudinal changes in these clinical and PROM-based measures on the brain age gap would also provide valuable insights into personalized MS management. In addition, multi-voxel MR spectroscopic imaging, together with voxel-wise mapping of microstructural metrics, could resolve regional metabolic and microstructural abnormalities across cortical gray matter, deep gray matter, and individual white-matter tracts, and relate these spatial patterns to the brain age gap and domain-specific cognitive dysfunction, thereby revealing circuit-specific mechanisms of accelerated aging in MS.

## 5. Conclusions

In conclusion, our study confirms that multiple sclerosis is associated with significantly accelerated biological brain aging, derived predominantly from a relapsing-remitting cohort. Crucially, we demonstrate that the brain age gap in MS extends beyond macroscopic atrophy, serving as a robust proxy for underlying subclinical metabolic failure and widespread microstructural degradation in normal-appearing tissues. This positions the brain-predicted age metric as a highly sensitive, multi-dimensional biomarker for monitoring invisible neuroaxonal pathology and evaluating therapeutic efficacy in clinical practice.

## Figures and Tables

**Table 1:** Demographic and clinical characteristics of the study population.

Characteristic	Total (N = 157)	Healthy Controls (N = 33)	MS Patients (N = 124)	*p*-Value
**Age (** **years** **)**	39.7 ± 11.2 *	30.0 [27.0–40.0]	39.0 [32.0–48.0]	**<0.01**
**Gender, No. (** **%** **)**				0.70
Female	102 (65.0%)	20 (60.6%)	82 (66.1%)	
Male	55 (35.0%)	13 (39.4%)	42 (33.9%)	
**BMI (** **kg/m^2^** **)**	28.8 (6.7) *	26.9 [22.9–29.8]	27.7 [24.0–34.0]	0.26
**Race, No. (** **%** **)**				**<0.01**
White	78 (49.7%)	16 (48.5%)	62 (50.0%)	
African American	68 (43.3%)	9 (27.3%)	59 (47.6%)	
Other	11 (7.0%)	8 (24.2%)	3 (2.4%)	
**Smoking Status, No. (** **%** **)**				**0.01**
Non-smoker	81 (60.0%)	13 (100.0%)	68 (55.7%)	
Smoker/Ex-smoker	54 (40.0%)	0 (0.0%)	54 (44.3%)	
**Alcohol use, No. (** **%** **)**				0.07
No		13 (100.0%)	85 (69.7%)	
Yes		0 (0.0%)	32 (26.2%)	
occasional		0 (0.0%)	5 (4.1%)	
**Clinical Course**				
RRMS	114 (94.2%)	—	114 (94.2%)	—
Other (SPMS/PPMS)	7 (5.8%)	—	7 (5.8%)	—

Values are expressed as median [interquartile range] or No. (%) unless otherwise indicated. Percentages represent valid cases, excluding missing data. * Overall mean (SD) provided for Age and BMI in the total column per baseline data.

**Table 2:** Comparison of MRI volumetric and microstructural metrics.

MRI Parameter	Healthy Controls (N = 33) Median [IQR]	MS Patients (N = 124) Median [IQR]	*p*-Value
**Brain Age Metrics**			
Brain Predicted Age (years)	31.8 [26.2–39.6]	53.3 [44.6–64.6]	**<0.001**
Age Gap (Predicted–Chronological)	−0.1 [−2.7–2.9]	10.2 [3.8–21.7]	**<0.001**
**Volumetric Measures (** **ml** **)**			
Grey Matter Volume	699.4 [654.4–745.9]	615.5 [573.1–662.1]	**<0.001**
White Matter Volume	475.3 [432.0–504.0]	437.8 [409.5–471.8]	**0.004**
CSF Volume	247.7 [204.7–288.1]	287.5 [230.5–364.4]	**0.002**
Total Deep GM Volume	51.4 [48.7–53.2]	42.5 [39.0–46.4]	**<0.001**
Global Cortical Thickness (mm)	2.5 [2.5–2.6]	2.4 [2.3–2.5]	**<0.001**
Choroid Plexus Volume (ChPV/TIV)	0.8 [0.6–1.1]	0.9 [0.7–1.1]	0.097
**Microstructural Integrity**			
NAGM MTR (%)	49.7 [49.4–50.6]	48.9 [47.8–49.7]	**0.006**
NAWM MTR (%)	57.1 [56.5–57.5]	56.5 [55.6–57.3]	**0.006**
NAGM Fractional Anisotropy	0.2 [0.2–0.3]	0.2 [0.2–0.2]	**0.005**
NAWM Fractional Anisotropy	0.5 [0.4–0.5]	0.4 [0.4–0.4]	**0.031**
tNAA/tCr Ratio	2.4 [2.3–2.6]	2.0 [1.8–2.2]	**<0.001**
NAGM Mean Diffusivity	0.9 [0.9–1.0]	1.0 [0.9–1.0]	**0.024**
NAWM Mean Diffusivity	0.8 [0.7–0.8]	0.8 [0.7–0.8]	0.745

Abbreviations: IQR, Interquartile Range; CSF, Cerebrospinal Fluid; GM, Grey Matter; WM, White Matter; NAGM, Normal Appearing Grey Matter; NAWM, Normal Appearing White Matter; MTR, Magnetization Transfer Ratio; tNAA/tCr, Total N-acetylaspartate to Total Creatine ratio. Sample sizes may vary across parameters owing to missing or non-evaluable data for some participants; reported values reflect available cases for each measure.

**Table 3:** Multivariable Linear Regression Models Predicting Brain Aging and Volumetric Outcomes.

Predictor	Estimate (B)	95% Confidence Interval	*p*-Value
**Model 1: Brain Predicted Age**	(R^2^ = 0.507)		
MS Diagnosis (vs. HC)	15.09	[10.66, 19.53]	**<0.001**
Chronological Age	0.65	[0.49, 0.80]	**<0.001**
Male Gender	1.33	[−2.19, 4.86]	0.456
Race (African American vs. White)	1.30	[−2.21, 4.81]	0.466
**Model 2: Brain Age Gap**	(R^2^ = 0.207)		
MS Diagnosis (vs. HC)	13.50	[8.84, 18.16]	**<0.001**
Male Gender	1.58	[−2.17, 5.33]	0.406
Race (African American vs. White)	2.07	[−1.65, 5.79]	0.273
**Model 3: Choroid Plexus Volume**	(R^2^ = 0.058)		
MS Diagnosis (vs. HC)	−0.05	[−0.19, 0.09]	0.526
BMI	−0.009	[−0.017, −0.001]	**0.023**
Male Gender	0.03	[−0.07, 0.13]	0.556
Age	0.002	[−0.003, 0.006]	0.454
Race (White vs. Other)	−0.09	[−0.36, 0.18]	0.509
Race (African American vs. Other)	−0.05	[−0.315, 0.219]	0.726

Note: Model 1 adjusted for Age, Gender, and Race. Model 2 adjusted for Gender and Race. Model 3 adjusted for Age, Gender, Race, and BMI.

## Data Availability

No patient data are shared in this paper. Requests from qualified investigators will be considered by the corresponding author in accordance with applicable privacy regulations.

## Abbreviations

The following abbreviations are used in this manuscript:

BA	Brain Age
BAG	Brain Age Gap
BICAMS	Brief International Cognitive Assessment for Multiple Sclerosis
BMI	Body Mass Index
ChPV	Choroid Plexus Volume
CSF	Cerebrospinal Fluid
CSI	Chemical Shift Imaging
CTh	Cortical Thickness
DMT	Disease-Modifying Therapy
DTI	Diffusion Tensor Imaging
EDSS	Expanded Disability Status Scale
EPI	Echo-Planar Imaging
FA	Fractional Anisotropy
FLAIR	Fluid Attenuated Inversion Recovery
FSL	FMRIB Software Library
GM	Grey Matter
GPR	Gaussian Process Regression
HC	Healthy Control
IQR	Interquartile Range
IRB	Institutional Review Board
LCModel	Linear Combination of Model spectra (spectroscopic analysis software)
MD	Mean Diffusivity
MPRAGE	Magnetization Prepared Rapid Gradient Echo
MRI	Magnetic Resonance Imaging
MRS	Magnetic Resonance Spectroscopy
MS	Multiple Sclerosis
MT	Magnetization Transfer
MTI	Magnetization Transfer Imaging
MTR	Magnetization Transfer Ratio
NAA	N-acetylaspartate
NAAG	N-acetylaspartylglutamate
NAGM	Normal-Appearing Grey Matter
NAWM	Normal-Appearing White Matter
nCPV	Normalized Choroid Plexus Volume
PPMS	Primary Progressive Multiple Sclerosis
PRESS	Point-Resolved Spectroscopy
PRL	Paramagnetic Rim Lesions
PROM	Patient-Reported Outcome Measures
RRMS	Relapsing-Remitting Multiple Sclerosis
SPM	Statistical Parametric Mapping
SPMS	Secondary Progressive Multiple Sclerosis
T2LV	T2 Lesion Volume
TIV	Total Intracranial Volume
tCr	Total Creatine (Creatine + Phosphocreatine)
tNAA	Total N-acetylaspartate (NAA + NAAG)
VIF	Variance Inflation Factor
VOI	Volume of Interest
WM	White Matter
RF	Radiofrequency
TR	Repetition time
TE	Echo time
TI	Inversion time
SPM12	Statistical Parametric Mapping 12
HCs	Healthy controls
GRE	Gradient-recalled echo
ppm	Parts per million
